# Feasibility and validation of a web-based platform for the self-administered patient collection of demographics, health status, anxiety, depression, and cognition in community dwelling elderly

**DOI:** 10.1371/journal.pone.0244962

**Published:** 2021-01-19

**Authors:** Matthew Calamia, Daniel S. Weitzner, Alyssa N. De Vito, John P. K. Bernstein, Ray Allen, Jeffrey N. Keller

**Affiliations:** 1 Department of Psychology, Louisiana State University, Baton Rouge, Louisiana, United States of America; 2 Pennington Biomedical Research Center, Baton Rouge, Louisiana, United States of America; University of New South Wales, AUSTRALIA

## Abstract

The coronavirus disease pandemic has brought a new urgency for the development and deployment of web-based applications which complement, and offer alternatives to, traditional one-on-one consultations and pencil-and-paper (PaP) based assessments that currently dominate clinical research. We have recently developed a web-based application that can be used for the self-administered collection of patient demographics, self-rated health, depression and anxiety, and cognition as part of a single platform. In this study we report the findings from a study with 155 cognitively healthy older adults who received established PaP versions, as well as our novel computerized measures of self-rated health, depression and anxiety, and cognition. Moderate to high correlations were observed between PaP and web- based measures of self-rated health (*r =* 0.77), depression and anxiety (*r =* 0.72), and preclinical Alzheimer’s disease cognitive composite (PACC) (*r =* .61). Test-retest correlations were variable with high correlations for a measure of processing speed and a measure of delayed episodic memory. Taken together, these data support the feasibility and validity of utilization of this novel web-based platform as a new alternative for collecting patient demographics and the assessment of self-rated health, depression and anxiety, and cognition in the elderly.

## Introduction

The coronavirus disease 19 (Covid-19) pandemic and the resulting direct and indirect impacts of social distancing dramatically interrupted or stopped clinical research around the world. These and other realities in the wake of Covid-19 have created a new urgency for the generation of web-based research platforms which provide alternatives to face-to-face and pencil-and-paper (PaP) based assessments, and reduce the dependence on the manual transfer of PaP data to an electronic database. Several computerized and web-based applications are currently available for conducting individualized assessments for a variety of clinical endpoints, including cognition [1]. However, these tools typically do not readily interact with a centralized study database and generally lack the ability to collect the supporting clinical data that accompany clinical research studies (e.g., demographics, secondary endpoint data collection). In order to address these research gaps, we have created a web-based platform which allows for the self-administered collection of patient demographics, the delivery and automated scoring of multiple assessments, and the capability to automatically populate all study data into a single secure and functional electronic database.

The fastest growing segment of the United States population is those 85 years of age and older, with age related diseases such as Alzheimer’s disease related dementia (ADRD) expected to increase from 5 million to 15 million in the next three decades [2, 3]. Recent ADRD research efforts have focused on developing multicomponent assessments of cognitive function, with an emphasis on developing composite cognitive assessments that are sensitive enough to measure the earliest changes relevant to the future development of ADRD. The Alzheimer’s Disease Cooperative Study Preclinical Alzheimer’s Cognitive Composite (ADCS-PACC) is a PaP based assessment package that has emerged as the leading clinical research tool for aging, mild cognitive impairment, and pre-ADRD research. The ADCS-PACC focuses on the assessment of the three cognitive domains which are the most predictive for the development of ADRD [4]. The ADCS-PACC is the primary endpoint in one of the largest clinical trials for AD prevention [5], and is a major cognitive endpoint for some of the largest longitudinal and cohort studies around the world [6]. Computer-based assessments have increasingly been used and valued for clinical care and research including studies of the elderly [7–9], clinical trials focused on cognition [10], and longitudinal studies with elderly participants [11, 12]. Currently there are no computerized/web-based options for the ADCS-PACC even though such an advance would provide a potential option that decreases the need for face-to-face assessments, manual scoring, manual z-score transformation, and manual data transfer to an electronic database that currently accompanies all ADCS-PACC efforts.

The current study focused on the validity and feasibility of using the computerized PACC (cPACC), a novel web-based application which employs a self-administered approach for elderly participants to provide demographic data as well as undergo assessments of self-rated health, depression, anxiety, and cognition. Analysis of 155 community dwelling elderly subjects demonstrates the feasibility of collecting data for each of these aspects in a self-administered manner that resulted in the automated population of a single, secured, cloud-based database. We report on the validity for each of the web-based measures with traditional PaP based assessments and report on their reliability as part of a two-week test-retest design in a subset of participants.

## Methods

### The demographic, assessment, and database platform

The platform used in this study was created by developers at Pennington Biomedical Research Center. The platform consisted of a web application written in Angular v.6 communicating with an API developed in Microsoft ASP.NET Core v2.1. The participants used the web-based application to answer a series of questions, and complete the different cognitive tasks, in a self-guided manner. As each question and assessment was completed the resulting data populated a central database that contained the demographic profile and assessment scores for each participant. All data was stored in a Microsoft SQL Server database. The entire system was operated as a web application in Microsoft Azure. Data was extracted from Azure using Microsoft SQL Server Management Studio. Administrative rights within the platform were used to control access to functionality and data.

### Participants

Individuals were recruited to the study who were 55–95 years old (inclusive), who did not have motor or sensory deficits that were sufficient to interfere with the ability of the participant to complete computerized assessments. Fliers, email blasts to a clinical registry of individuals aged 50 and over, and word of mouth were used to recruit participants in the Baton Rouge area. A total of 174 participants met study criteria and provided written informed consent. Most of these participants (n = 155) had Mini-Mental State Examination (MMSE) scores in a range suggesting intact global cognition (i.e., greater than or equal to 25) and are the focus of all analyses other than the one analysis also comparing their scores against a small group of participants with MMSE scores below 25 (n = 19). Demographic information for the study sample is provided in Tables 1 and 2. See Table 3 for raw performance data for participants. Missing data ranged from 0–15 participants across measures.

**Table 1:** Participant demographics.

	MMSE ≥ 25		MMSE < 25	
*Demographic Variables*	n (%)	Mean (SD)	n (%)	Mean (SD)
Age	-	71.64 (8.13)	-	75.94 (11.10)
Female	111 (71.6%)	-	6 (31.6%)	-
Non-Hispanic	145 (93.5%)	-	18 (94.7%)	-
*Race*				
Caucasian	140 (90.3%)		17 (89.5%)	-
African American	7 (4.5%)	-	1 (5.3%)	-
Bi-racial	2 (1.3%)	-	-	-
Native American	1 (0.01%)	-	-	-
*Highest Degree of Education*				
GED	10 (6.5%)	-	1 (5.3%)	-
Some College	33 (21.3%)	-	1 (5.3%)	-
Associate’s Degree	7 (3.9%)	-	1 (5.3%)	-
Bachelor’s Degree	41 (26.5%)	-	7 (36.8%)	-
Master’s Degree	52 (33.5%)	-	4 (21.1%)	-
Doctorate Degree	6 (3.9%)	-	2 (10.5%)	-
*Marital Status*				
Married	82 (55.0%)		9 (47.4%)	-
Widowed	28 (18.8%)		6 (31.6%)	-
Divorced	23 (15.4%)		2 (10.5%)	-
Never Married	14 (9.4%)		1 (5.3%)	-
Common-Law Partner	2 (1.3%)		-	-
*Living Situation*				
Living Alone	48 (31.0%)		5 (26.3%)	-
*Residence Type*				
Single Family Home	111 (71.6%)		11 (57.9%)	-
Apartment	35 (22.6%)		4 (21.1%)	-
Assisted Living	3 (0.6%)		3 (15.8%)	-

Note: Demographic information for some variables was unavailable and therefore not all variables will sum to a total of 155 and 19 individuals. MMSE = Mini-Mental State Examination

**Table 2:** Prevalence of health conditions in entire sample.

Health Condition	n (%)
*Cardiovascular*	
High Blood Pressure	72 (46.5%)
High Cholesterol	55 (35.5%)
Diabetes	18 (11.6%)
Heart Attack	4 (2.6%)
Atrial Fibrillation	14 (9.0%)
*Neurological*	
Stroke	2 (1.3%)
Parkinson’s Disease	2 (1.3%)
Multiple Sclerosis	0 (0.0%)
Transient Ischemic Attack	1 (0.6%)
Alzheimer’s Disease	0 (0.0%)
Other Dementia	3 (1.9%)
Other Neurological Disease	4 (2.6%)
Concussion/TBI	2 (1.3%)
*Psychiatric*	
Alcohol Abuse	3 (1.9%)
Drug Abuse	1 (0.6%)
Depression	31 (20.0%)
Anxiety	27 (17.4%)
*Other*	
B12 Deficiency	6 (3.9%)
Sleep Apnea	17 (11.0%)
Thyroid Deficiency	33 (21.3%)
Cancer	26 (16.8%)

**Table 3:** Means and standard deviations for cognitive measures and questionnaires in participants scoring above and below 25 on the MMSE.

	MMSE ≥ 25				MMSE < 25			
*Variables*	n	Mean (*SD*)	Min	Max	n	Mean (SD)	Min	Max
FNHR-IFR	148	6.54 (*2*.*90*)	0	14	17	2.53 (*2*.*24*)	0	8
FNHR-IR	149	28.33 (*2*.*99*)	17	32	16	21.06 (*7*.*04*)	10	31
FNHR-DFR	148	10.24 (*3*.*52*)	2	16	15	4.27 (*4*.*85*)	0	14
FNHR-DR	149	14.65 (*2*.*01*)	0	16	15	10.00 (*3*.*70*)	4	16
GLIR	155	14.05 (*3*.*74*)	5	23	16	8.44 (*3*.*98*)	4	18
GLDR	151	7.03 (*2*.*67*)	0	12	19	3.53 (*2*.*95*)	0	10
SL	149	10.50 (*6*.*44*)	0	24	18	4.00 (*3*.*93*)	0	14
SM	140	24.54 (*7*.*85*)	0	42	16	11.44 (*7*.*25*)	0	24
VP	142	19.27 (*5*.*58*)	1	28	16	9.63 (*7*.*16*)	0	19
VAS	152	83.91 (*13*.*33*)	18	100	18	82.33 (*12*.*98*)	60	100
LM-DR	152	6.19 (*3*.*02*)	1	18	17	2.24 (*2*.*93*)	0	8
DSC	151	51.11 (*13*.*49*)	24	90	17	28.06 (*13*.*83*)	3	64
FCSRT	151	47.66 (*1*.*48*)	31	49	17	40.24 (*10*.*83*)	4	48
GAI	152	1.61 (*3*.*06*)	0	16	18	3.00 (*2*.*68*)	0	10
GDS	152	5.54 (*4*.*84*)	0	25	17	8.18 (*5*.*87*)	1	22

Note: SD = Standard Deviation; MMSE = Mini-Mental State Examination; FNHR-IR = Face Name Hobby Recall Immediate Free Recall; FNHR-IFR = Face Name Hobby Recall Immediate Recognition; FNHR-DFR = Face Name Hobby Recall Delayed Free Recall; FNHR-DR = Face Name Hobby Recall Delayed Recognition; GLIR = Grid Locations Immediate Recall; GLDR = Grid Locations Delayed Recall; SL = Symbol Line; VP = Visual Patterns; SM = Speeded Matching; VAS = EQ-5D Visual Analog Scale; Logical Memory–Delayed Recall; DSC = Digit Symbol Coding; FCSRT = Free and Cued Selective Reminding Test; GAI = Geriatric Anxiety Inventory; GDS = Geriatric Depression Scale.

### Procedures

The measures were administered on the same day, with half of the participants completing the PaP measures first, and the other half completing the web-battery first. Participants completed the measures in a quiet and private testing room on either a desktop or laptop computer with a computer mouse. PaP measures were administered by a trained research assistant. Research assistants remained in the room while participants completed the computerized measures, but only to address technological issues (e.g., computer froze/internet connection issues) or provide encouragement to participants.

A subset of the sample with MMSE scores greater than or equal to 25 (n = 55) were randomly selected to complete a second visit approximately two weeks later during which they repeated the cPACC to assess for test-retest reliability. The first study visit was on June 11, 2018 and the last study visit was on October 9, 2019. All study procedures were approved by the LSU Institutional Review Board and were conducted according to the principles expressed in the Declaration of Helsinki. All data collected during the assessment were stored immediately at the conclusion of each page. Data were written to a Microsoft SQL Server 2014 database and stored as the raw answer provided by the participant. Answers to some tests such as the participant typing the name and hobby of a person in an image were reported as the exact text entered by the participant. Other tests using multiple choice answers or clicks on a grid were scored as number of correct answers and where applicable number of attempts.

### Paper and Pencil (PaP) measures

#### Questionnaires

Participants completed the EQ-5D Visual Analog Scale (VAS) to assess self-rated health [13]. For this measure, participants rate their current health on a 0 to 100 scale from the “worst health” to “best health” they can imagine. The EQ-5D VAS is sensitive to individual differences such as age [14] and physical activity [15]. The Geriatric Anxiety Inventory (GAI) and Geriatric Depression Scale (GDS) were used to assess anxiety and depression, respectively. The GAI is a 20-item geriatric-focused self-report measure of anxiety-related symptoms [16]. The GAI demonstrates excellent internal consistency (α = 0.91) and test-retest reliability (r = 0.91) [16] as well as good convergent validity with worry and anxiety measures [17]. The Geriatric Depression Scale (GDS) is a 30-item self-report which measures depressive symptoms in older adults [18]. The GDS demonstrates excellent internal consistency (α = 0.94), good test-retest reliability (r = 0.84) [19], and at least adequate convergent validity with other depression measures such as the Beck Depression Inventory-II (r = .78) [20]. However, despite good convergent validity, the discriminant validity of these measures is weak with one study finding a correlation as high as r = .86 between the GAI and GDS [21]. A 12-item computer proficiency questionnaire [22] was used in order to assess how easily older adults felt they could perform tasks on a computer (e.g., “Use a keyboard to type”) in a 5-point likert scale format. The sample had a self-reported mean computer proficiency rating of 3.17 (*SD =* .*98*), indicating that on average, they could somewhat easily perform computer-based tasks.

*Alzheimer’s Disease Cooperative Study Preclinical Alzheimer’s Cognitive Composite (ADCS-PACC)*. A review by Alzheimer’s disease cooperative study (ADCS) identified episodic memory, executive function, and orientation as the 3 key cognitive domains linked to the development of mild cognitive impairment and ADRD [4]. A total of 4 pencil-and- paper (PaP) cognitive assessments were selected to capture these domains as part of the ADCS Preclinical Alzheimer’s Disease Composite (PACC). The ADCS-PACC is comprised of immediate recall on the Free and Cued Selective Reminding Test (FCSRT), delayed recall on one story from the Logical Memory subtest of the Wechsler Memory Scale-Revised battery, the Digit-Symbol Test from the Wechsler Adult Intelligence Scale-Revised, and the MMSE.

Studies using the ADCS-PACC have been able to identify study subjects who would go on to develop clinical biomarkers of ADRDs such as pathological beta amyloid deposition as well as identify which study subjects exhibit the fastest rate of cognitive decline in longitudinal studies [23, 24]. Due to these successes, the ADCS-PACC has emerged as one of the most commonly utilized cognitive batteries in prominent clinical trials and longitudinal research studies including the A4 trial and Alzheimer’s Disease Neuroimaging Initiative (ADNI), respectively.

### Web-battery measures

#### Questionnaires

The web battery included survey items regarding participant demographics (i.e., date, gender, zip code, ethnicity, race, marital status, living situation, and highest level of education attained), and health history (i.e., a list of conditions presented as a checklist). Additionally, we collected information on family history of dementia, pain severity and interference on daily functioning, frequency of exercise, number of medications and medication adherence, concern about driving and accident history, self-rated health, and subjective memory complaints that will be a part of future research. Responses to the demographic and health history questions can be found in Tables 1 and 2.

For the purposes of this study, psychometric validation focused on 1) a self-report measure of health in which participants make one global rating of their health and 2) a new 17-item measure of depression and anxiety developed based on widely used measures of depression and anxiety. Participants were asked to rate how much they felt or experienced certain symptoms over the past 2 weeks on a 5-point scale (“not at all” to “extremely”). Given that brief measures of depression and anxiety show poor discriminant validity [15], these symptoms were assessed jointly rather than with the aim of developing two separate scales.

*Computerized Preclinical Alzheimer’s Cognitive Composite (cPACC)*. The cognitive measure in this study was a cognitive composite that was validated against the (ADCS-PACC). Like the ADCS-PACC, the cPACC was designed to assess the domains of orientation and episodic memory. The cPACC also includes a measure of processing speed designed to be comparable to the PaP measure of digit symbol coding which the PACC considers a measure of executive functioning. Additionally, the cPACC includes measures of working memory given working memory is related to executive functioning [25], a PACC domain, and a working memory item is included on the MMSE which is used as part of the PACC.

*Orientation*. For orientation participants are asked orientation questions on the computer screen (day, year, time of day, etc.) and select the answers from a list of multiple-choice response options. Participants receive 1 point for each correct answer.

*Face Name Hobby Recall (FNHR)*. This cPACC component is designed to assess episodic memory which is one component of the ADCS-PACC. It is based on the short version of the Face-Name Associative Memory Exam [26–28]. For cPACC Faces and Names the participant first completes a learning trial in which 8 faces with a name and hobby presented underneath. The names and hobbies chosen are short in word length (e.g., Amy, Hiker). Faces vary in age, gender, and race. Stimuli are presented twice and are followed by immediate recall trial each time in which they have to recall the names and hobbies when presented only with the face by typing their responses into a text box and then clicking “next” to submit their response. Participants receive 1 point each for correctly naming the person’s hobby and their name, for a total of 2 points per stimulus. An immediate recognition trial then follows in which they must select the correct name and hobby from a multiple-choice list by clicking on the correct stimulus. Participants receive 1 point for correctly selecting each name and hobby for a total of 2 possible points. After a ~15-minute delay in which they complete other cPACC measures, delayed recall and recognition trials are completed

*Grid locations*. The grid location test is a measure of visual episodic memory designed based on the Visual Spatial Learning Test [29, 30], a measure designed to be a visual equivalent to verbal list-learning paradigms. Scores on this measure highly correlated with verbal memory measures [29, 30]. For cPACC grid locations, participants complete two learning and immediate memory trials in which they see 6 symbols on a 4x4 grid and then have to select the symbols they saw and put them in the correct location. For each symbol, participants can earn up to 2 points (1 point for selecting the correct symbol and 1 point for placing the symbol in the correct location), for a possible of 12 points. The same symbols and locations are used for both learning trials. Participants then complete a delayed memory trial after ~15-minute delay.

*Speeded matching*. Speeded matching is a measure of processing speed and executive function that is based on the Wechsler Adult Intelligence Scale—Revised (WAIS-R) Digit Symbol Coding subtest [31], a measure included in the ADCS-PACC. For the cPACC participants have 90 seconds to select symbols that correspond to numbers based on a key matching each unique symbol to a specific number. As participants select symbols, they appear in the blank boxes above the numbers. As participants complete more matches, additional numbers with blank boxes above them appear on the screen. Participants receive 1 point for each correctly selected symbol.

*Symbol line*. Symbol line is a measure of visual working memory based on Wechsler Memory Scale—Fourth Edition (WMS-IV) Symbol Span [32]. Participants see a line of symbols and then have to correctly select which symbols they saw in the correct order (i.e., left to right). Initially participants are shown only two symbols in a line, but lines of increasing lengths are added until a participant makes no correct responses or is presented with a trial of 7 symbols. For each symbol, participants can achieve a total possible of 2 points. If participants recall incorrect symbols, they receive 0 points. If all of the correct symbols are recalled, but in the incorrect order, participants receive 1 point. If participants recall the correct symbols in the correct order, they receive 2 points.

*Visual patterns*. Visual patterns is a measure of visual working memory based on Wechsler Memory Scale - 3rd Edition (WMS-III) Spatial Span [33]. Participants see an array of 9 white boxes and are asked to recall the order in which boxes are turned black. A box that is turned black returns to white before the next box turns black. Initially participants are shown only two boxes that are turned black but increasing numbers of boxes are turned black until a participant makes no correct responses or is presented with a trial of 7 boxes. Participants receive 1 point for the correct completion for each sequence.

### Analyses

#### Validity

Pearson correlations were used to examine the relationship between scores on questionnaire measures administered via PaP or the web-battery. For the GAI and GDS, scores were first converted to z-scores and a composite was created to compare with the web-based measure of depression and anxiety symptoms. For the web-battery measure of depression and anxiety, a confirmatory factor analysis (CFA) was first conducted as part of assessing construct validity to assess whether a one-factor model provided adequate model fit. To compare the ACDS-PACC and cPACC using a Pearson correlation, individual tests administered were also first converted to z-scores. Thus, for the PaP, the FCSRT, Logical Memory Delayed Score, and MMSE total score were individually standardized into Z-scores, and then summed together to create the PaP composite score. To create the cPACC composite score, the number of correct responses recalled during the Faces and Names immediate and long-delay free recall and multiple choice, Grid Locations, Symbol Matching, Symbol Line, and Grid Pattern tasks were individually standardized into Z-scores. These Z-scores were then summed together to create the cPACC composite score. Only two participants in our cognitively intact sample missed an orientation item. Therefore, orientation was not included when creating a cPACC composite score.

To assess the sensitivity of the cPACC to cognitive impairment, we calculated effect sizes using Hedges’ *g* to determine whether the subtests of the cPACC could differentiate between those with MMSE scores above and below 25. Hedges’ *g* was used given the large difference in sample sizes between the cognitively healthy and cognitively impaired group.

#### Practice effects

Dependent t-tests and Cohen’s *d* were used to examine practice effects on the web-battery in the subsample who completed a second visit approximately two weeks following the initial visit.

#### Reliability

To assess internal consistency of the measure of depression and anxiety, coefficient alpha was used. To assess the test-retest reliability of the web-battery, Pearson correlations were used to examine the relationship of questionnaire and cognitive test scores administered within an approximately two-week test-retest interval.

## Results

### Validity and reliability of the web-based questionnaire

**Fig 1:**
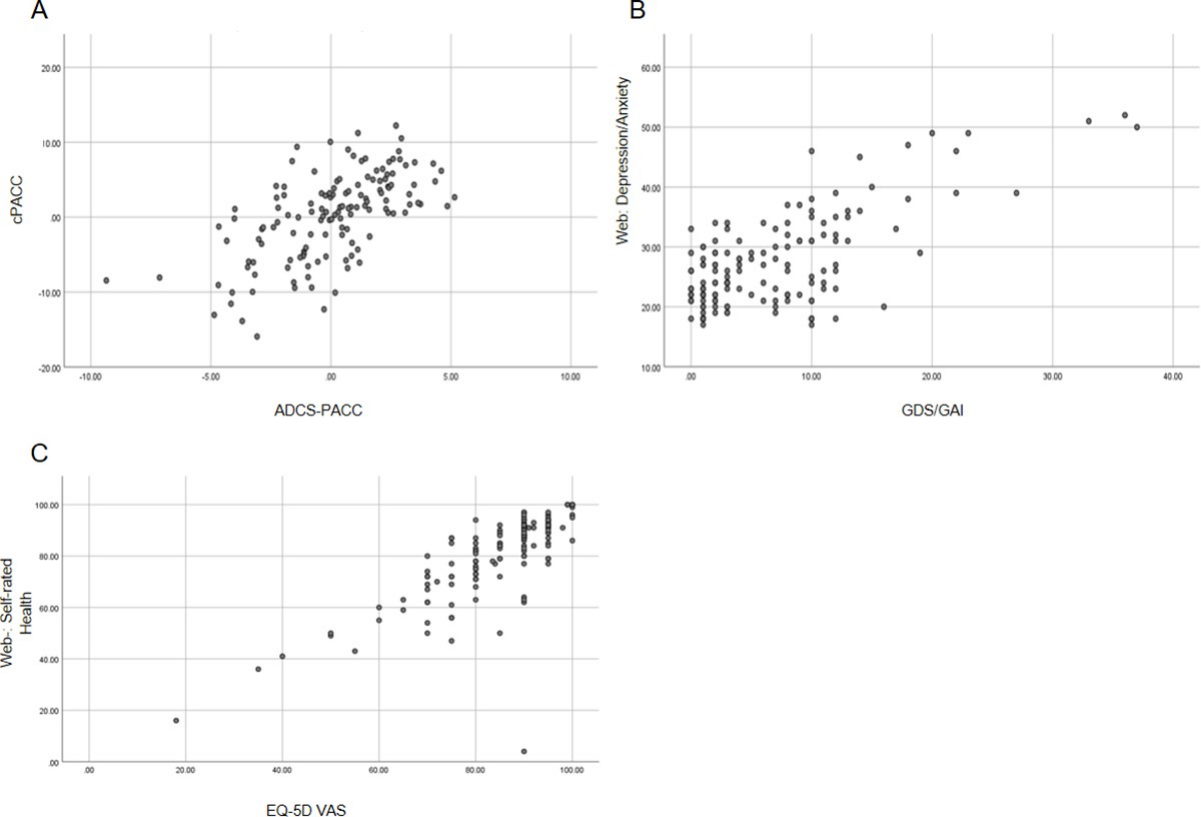
Relationships between web-based measures and paper and pencil measures. Note: A.) Relationship between the Computerized Preclinical Alzheimer’s Cognitive Composite (cPACC) and the Alzheimer’s Disease Cooperative Study Preclinical Alzheimer’s Cognitive Composite (ADCS-PACC). B.) Relationship between the web-battery measure of depression and anxiety and the GDS/GAI composite score. C.) Relationship between the web-battery measure of self-rated health and the EQ-5D Visual Analog Scale (VAS).

Adequate fit for a one-factor model for the web-battery measure of depression and anxiety (CFI = 0.91, RMSEA = 0.08) was obtained when allowing for two pairs of correlated residuals for items with similar content (i.e., “I was easily upset” and “I was easily annoyed”; “I had difficulty stopping myself from worrying” and “I worried a lot.”). Coefficient alpha for this scale was .91. A high correlation was observed between the web-battery measure of depression and anxiety and the GDS/GAI composite, r = 0.70. Similarly, a high correlation, r = .77, was obtained between the web-battery measure of self-rated health and the EQ-5D VAS (see Fig 1).

In the 55 older adults in the sample who completed a retest after approximately 2 weeks, the web-battery measure of depression and anxiety and self-rated health both had high test-retest correlations, *r* = 0.85 and *r* = .0.83, respectively.

### Validity and reliability of the Computerized Preclinical Alzheimer’s Cognitive Composite (cPACC)

The composite scores derived from the cPACC and the ADCS-PACC were found to be moderately related (*r =* .61) (Fig 1). As an additional exploratory analysis using stepwise regression showed that this same correlation could be obtained using only a subset of measures: Speeded Match, the immediate trials of Face Name Hobby Recall, the immediate trials of Face Name Hobby Recognition, and the delayed trial of Grid Locations (*F*(4,132) = 22.08, R^2^ = .401). See S1 Table for the full results of the regression analysis. In addition, the Speeded Match task moderately correlated to the Digit Symbol Coding subtest (*r* = .56), thus demonstrating convergent validity between a measure of the cPACC and a PaP measure it was designed to match (see S2 Table for relationships among all measures on the CPACC and the PaP measures).

All of the measures of working memory, episodic memory, and processing scored as part of the cPACC battery significantly differed (Hedges’ g ranged from 1.12 to 2.30) between those above and below an MMSE score of 25, which is a common cutoff for cognitive impairment. The differences between those above and below an MMSE score of 25 were larger in the composite PaP score compared to the composite cPACC score (Hedges’ *g* = 2.98 vs 2.31). However, when the MMSE was removed from the PaP composite score, the differences between those above and below an MMSE score of 25 were larger in the composite cPACC score compared to the composite PaP score (Hedges *g* = 2.31 to 2.17).

High test-retest reliability was obtained on delayed free-recall and multiple-choice subtests of the Faces and Names test (*r* = .70 to *r* = .74) as well as on a measure of processing speed similar to digit symbol coding on the PaP (*r* = .73) (Table 4). However, tasks of visual working memory demonstrated weak to moderate test-retest reliability (*r* = .36 to *r* = .45).

**Table 4:** Test-retest correlations and practice effects between baseline and follow-up visits.

Test	R	*t*	*d*
Face Name Hobby Recall Immediate Free Recall	.56	9.52***	1.25
Face Name Hobby Recall Immediate Recognition	.59	6.34***	.78
Face Name Hobby Recall Delayed Free Recall	.70	5.84***	.61
Face Name Hobby Recall Delayed Recognition	.74	3.93***	.39
Grid Locations Immediate Recall	.57	6.25***	.78
Grid Locations Delayed Recall	.48	3.60**	.49
Symbol Line	.36	1.08	.16
Visual Patterns	.45	1.37	.19
Speeded Matching	.73	.075	.02

Note: All correlations significant at *p* < .01

** indicates significant dependent t-test value at the *p* < .01 level

*** indicates significant dependent t-test value at the *p* < .001 level

*d* = Cohen’s d

Both episodic memory tasks demonstrated significant practice effects (*p*’s < .01) on both immediate and delayed-recall trials. However, measures of processing speed and visual working memory tasks did not (*p*’s > .05; *see* Table 4).

## Discussion

The current study demonstrates the feasibility of using a novel web-based application for the collection of study subject demographics, as well as the results from diverse computer-based assessments, in the elderly. The current feasibility and validity study was conducted under conditions where data was collected both in traditional research settings (i.e., lab space on a university campus) as well as in senior living communities. Although a research assistant was present in case assistance was needed for participants to navigate the web-battery, for nearly all participants the interaction was primarily limited to providing encouragement during testing. Encouragement was needed in large part due to the fact the participants completed extensive PaP as well as web-based battery in the same day. In a minority of participants assistance with using the computer and/or providing further clarifications to the questions and tasks that were being asked. In future studies it will be important to further refine the delivery of the web-based assessments in order to minimize/eliminate the involvement of research personnel in the evaluation. Exploratory step wise regression analysis identified that the use of a greatly abbreviated cPACC battery was sufficient to capture the observed validity between cPACC and ADCS-PACC (Speeded Match, FNHR, Grid Locations). Together these observations point to the ability to reduce or eliminate participant frustration by using an abbreviated cPACC and/or minimizing the amount of PaP assessments in future validation efforts.

Although further validation is needed, one potential use for this platform is to provide an option for the self-administered collection of assessments and patient demographics in a clinical setting that involves little to no involvement of clinical staff. Additionally, in the current study use of this web-based platform occurred in some instances in assisted-livings raising the potential for conducting evaluations outside of traditional clinic setting, including an individual’s home. Both the limited involvement of clinical staff and ability to administer evaluations outside of the traditional setting are increasingly important aspect of clinical research given the impacts of Covid-19.

We observed that multiple assessments within the current platform provided valid measures for diverse aspects of geriatric health. Specifically, we identified the ability of the platform to capture self-reported patient demographics as well as valid measurements self-rated health, depression and anxiety symptoms in a sample of community dwelling elderly. The relationship that was observed between the web-battery measure of depression and anxiety and the GDS/GAI composite in the current study was similar to correlations found in other studies reporting measures of depression and anxiety (e.g., [34–36]). It is important to point out that the platform therefore not only contains cognitive assessments but also includes other endpoints that are routinely required as part of cognition focused studies.

There is a widespread and growing use of the ADCS-PACC in clinical trials and longitudinal studies, and therefore there is a need to produce ADCS-PACC assessment options that don’t require traditional PaP delivery/capture during periods of significant operational and safety challenges such as Covid-19. We developed the current web-based battery to provide a mechanism to capture an ADCS-PACC relevant assessment that could be delivered using a computer-based application in place of a PaP. While our computer-based assessment taps into cognitive domains relevant to the ADCS-PACC, and significantly correlates with performance on a PaP version of the ADCS-PACC (moderate significance), we recognize that there are verbal and mechanical limitations in the current computer-based assessment does not allow for a complete overlap with the individual assessments comprising the ADCS-PACC. Further, the tests that comprise the computer-based assessment were designed to address similar constructs to the ADCS-PACC but the format and demands are different even for tests most similar to one another. For example, orientation was asked using multiple choice questions on the cPACC while the MMSE asks for a verbal response without cues and the digit symbol coding requires written copy of symbols while the speeded match task involves using a mouse to click on a response. In this initial validity study, we identified the cPACC to have a statistically significant (moderate correlation) with the PaP version of the ADCS-PACC, and to have comparable discrimination to the ADCS-PACC in terms identifying those with and without cognitive impairment. Interestingly, when the MMSE was removed from the PaP composite score, the cPACC was better able to distinguish between those with and without cognitive impairment. To our knowledge, there has only been one previous validation study of computer-based assessments targeting the ADCS-PACC [10]. That study demonstrated that the computerized batteries had positive correlations with the ADCS-PACC. Therefore, the results of the current study add to a limited, but growing literature which represents a potentially important step in moving from a reliance upon PaP versions of the ADCS-PACC for the measurement of an ADRD relevant cognitive composite. In particular, it will be important to determine in the near future the ability to extend the findings from this initial validation study to a larger and more diverse study sample that also includes data as to the feasibility of using the cPACC for measuring the rates of cognitive change over time.

Inherent cPACC features such as the automated assessment delivery and scoring may facilitate cognitive composite measures being conducted in a larger number of clinical and research settings. The cPACC demonstrated good reliability when assessing delayed memory both through free recall and when given further cuing through multiple choice on the FHNR test. To our knowledge this is the first study to describe the use of a recall component in a computerized episodic memory test. The FHNR task is based on the Face-Name Association Memory Test which has been shown to distinguish between cognitive healthy individuals and those with MCI and correlates with AD biomarkers such as amyloid deposition [26, 28]. Given the size of practice effects observed for episodic memory measures, a future goal is to develop alternate forms to reduce practice effects.

In addition to verbal episodic memory, visual episodic memory has shown to decline in a similar magnitude in individuals at risk for ADRD [37] and visual episodic memory measures cognitive impairment beyond verbal episodic memory alone [38]. Measures of visual episodic memory and visual working memory are extremely feasible and conducive for a computer-based delivery of cognitive assessments and are components of the cPACC [39, 40]. However, with the exception of a task assessing processing speed, all other tests demonstrated weak to moderate test-retest correlations in the current study. One possible solution to improve the test-retest reliability of the cPACC is to add more trials to the visual episodic memory tests. Despite this, subtests of the cPACC demonstrated strong effect sizes in distinguishing between those with and without subtle cognitive impairment. Future studies can explore the ability of the cPACC to identify subtle cognitive impairments in older adults.

Participants in the current study did not demonstrate variability in responses to the orientation items (only 3 participants in the entire sample did not get both orientation questions correct). For the PACC, the MMSE is included given it includes items to measure orientation, a domain identified in the review as important for assessing preclinical AD. However, the MMSE, is known to have poor psychometric properties (i.e., ceiling effects and low test-retest-reliability) in healthy, non-demented, older adults [41]. In some circumstances removing the MMSE has actually been shown to improve the sensitivity of the ADCS-PACC to measure cognitive decline [42]. Taken together, these data highlight the importance of the need to continue to optimize the psychometric properties of the ADCS-PACC.

A number of studies have identified important roles of working memory in the development of MCI and progression to ADRD. Modifications to the ADCS-PACC which add in a measure of verbal fluency, tasks highly linked to working memory, were found to be better than the original PACC in capturing longitudinal decline [42, 43]. Verbal fluency measures can be considered as measures of executive functioning, a domain identified as important in the assessment of preclinical AD [44]. While verbal fluency measures are difficult to incorporate into a computerized testing setting, visual working memory measures can be readily implemented in a computer-based assessment and are sensitive to the identification of cognitive decline associated with ADRD [45]. Further validation efforts and implementation of the cPACC may identify that it has enhanced sensitivity and utility with which to measure and monitor cognitive change relevant to the development of ADRD.

The focus of the current study was to conduct an initial validation study of the web-battery, including the cPACC in a non-demented, community dwelling, sample of older study participants. Of note, only a subset of the much larger web-battery questionnaire was the focus of psychometric validation and future studies will need to validate the remaining questions. A limitation of the current study is observed in the study sample being overwhelmingly Caucasian and well-educated which is not representative of the general population raises caution in extending the findings from this study to a more ethnically and educationally diverse sample. Validation of the cPACC was based on cross sectional data and caution should be applied in determining the ability of the cPACC to measure cognitive change in a longitudinal manner similar to previous studies reported with the ADCS-PACC. Further, in making comparisons between those with intact global cognition (i.e., MMSE score of 25 or higher) and reduced global cognition (i.e., MMSE score less than 25), the current study had a small number of participants with reduced global cognition. Future studies can continue to examine the utility of the cPACC to differentiate between those with intact and reduced cognitive performance.

## Supporting information

S1 Table Stepwise regression of cPACC measures and the PaP composite score. (DOCX)

S2 Table Correlations among measures on the cPACC and PaP measures. (DOCX)
