# Supplementary material for: Feasibility and validation of a web-based platform for the self-administered patient collection of demographics, health status, anxiety, depression, and cognition in community dwelling elderly
Source: PLoS One. 2021 Jan 19;16(1):e0244962. doi: 10.1371/journal.pone.0244962 (PMC7815113; doi:10.1371/journal.pone.0244962)
Supplement: S1 Table — (DOCX) [file pone.0244962.s001.docx]

S1 Table. Stepwise Regression of cPACC Measures and the PaP Composite Score.

| Model 1 | | | | | |
| --- | --- | --- | --- | --- | --- |
| Predictor | B | SE | β | t | *p* |
| Constant | -11.61 | 1.77 | - | -6.57 | <.001 |
| FNHR-IR | .41 | .06 | .50 | 6.63 | <.001 |
| R² | .245 | | | | |
| Model 2 | | | | | |
| Predictor | B | SE | β | t | *p* |
| Constant | -11.28 | 1.66 | - | -6.78 | <.001 |
| FNHR-IR | .31 | .06 | .37 | 4.86 | <.001 |
| SM | .11 | .03 | .33 | 4.31 | <.001 |
| R² | .337 | | | | |
| Model 3 | | | | | |
| Predictor | B | SE | β | t | *p* |
| Constant | -12.20 | 1.66 | - | -7.36 | <.001 |
| FNHR-IR | .29 | .06 | .34 | 4.60 | <.001 |
| GLDR | .14 | .05 | .20 | 2.75 | .007 |
| SM | .09 | .02 | .27 | 3.57 | .001 |
| R² | .373 | | | | |
| Model 4 | | | | | |
| Predictor | B | SE | β | t | *p* |
| Constant | -1.24 | 1.81 | - | -5.66 | <.001 |
| FNHR-IFR | .18 | .07 | .21 | 2.48 | .015 |
| FNHR-IR | .18 | .07 | .22 | 2.51 | .013 |
| GLDR | .12 | .05 | .18 | 2.51 | .013 |
| SM | .09 | .02 | .27 | 3.53 | .001 |
| R² | .401 | | | | |

Note: B = Unstandardized coefficient; SE = Standard error; β = Standardized coefficient; FNHR-IR = Face Name Hobby Recall Immediate Free Recall; FNHR-IFR = Face Name Hobby Recall Immediate Recognition; GLDR = Grid Locations Delayed Recall; SM = Speeded Matching
