# Supplementary material for: Feasibility and validation of a web-based platform for the self-administered patient collection of demographics, health status, anxiety, depression, and cognition in community dwelling elderly
Source: PLoS One. 2021 Jan 19;16(1):e0244962. doi: 10.1371/journal.pone.0244962 (PMC7815113; doi:10.1371/journal.pone.0244962)
Supplement: S2 Table — (DOCX) [file pone.0244962.s002.docx]

S2 Table. Correlations Among Measures on the cPACC and PaP Measures.

| **Test** | FCSRT | LM | DS |
| --- | --- | --- | --- |
| Face Name Hobby Recall Immediate Free Recall | .207* | .244*** | .286*** |
| Face Name Hobby Recall Immediate Recognition | .219** | .292*** | .383*** |
| Face Name Hobby Recall Delayed Free Recall | .255** | .326*** | .305*** |
| Face Name Hobby Recall Delayed Recognition | .038 | .263** | .298*** |
| Grid Locations Immediate Recall | .250** | .139 | .302*** |
| Grid Locations Delayed Recall | .129 | .164* | .259** |
| Symbol Line | .144 | .042 | .359*** |
| Visual Patterns | .144 | .084 | .259** |
| Speeded Matching | .211* | .066 | .557*** |

Note: FCSRT = Free and Cued Selective Reminding Test; LM = Logical Memory subtest of the Wechsler Memory Scale-Revised; DS = Digit-Symbol Test from the Wechsler Adult Intelligence Scale-Revised.

All values represent Pearson correlations.

*indicates a significant correlation at the *p* < .05 level

** indicates a significant correlation at the *p* < .01 level

*** indicates a significant correlation at the *p* < .001 level
